# Supplementary material for: Extracellular rRNA Profiling Reveals the Sinking and Cell Lysis Dynamics of Marine Microeukaryotes
Source: Environ Microbiol. 2025 Aug 5;27(8):e70164. doi: 10.1111/1462-2920.70164 (PMC12324905; doi:10.1111/1462-2920.70164)
Supplement: Supplementary file 1 — Data S1: Supporting Information. [file EMI-27-e70164-s001.pdf]

Table S1 Geographic locations of sampling sites.

Station	Latitude (°N)	Longitude (°E)	Dampling date	Dampling time (JST)	Depth category	Depth (m)	Zone
3	28.00	128.50	22-October 2022	0:38	10 m	10	Epipelagic
					SCM	93	Epipelagic
					MES	300	Mesopelagic
E1	28.50	128.39	23-October 2022	21:40	10 m	10	Epipelagic
					SCM	54	Epipelagic
					MES	500	Mesopelagic
5	30.50	128.00	20-October 2022	23:45	10 m	10	Epipelagic
					SCM	53	Epipelagic
					MES	300	Mesopelagic
6	30.63	128.55	21-October 2022	23:43	10 m	10	Epipelagic
					SCM	35	Epipelagic
					MES	500	Mesopelagic
8	30.90	129.67	25-October 2022	9:22	10 m	10	Epipelagic
					SCM	43	Epipelagic
					MES	300	Mesopelagic

Table S2 Primer pairs used in this study.

Experiments/Primer name	Sequence (5' to 3')	References
***E. coli* strain B-specific 16S V4 primer**		
16S_EC442F	GCGGGGATGAAGGGAGTAAA	This study
16S_EC636R	ATGCAGTTCCCAGGTTGAGC	This study
**Eukaryote-specific 18S V4 primer**		
E572F	CYGCGGTAATTCCAGCTC	Comeau et al. (2011)
E1009R	AYGGTATCTRATCRTCTTYG	Comeau et al. (2011)

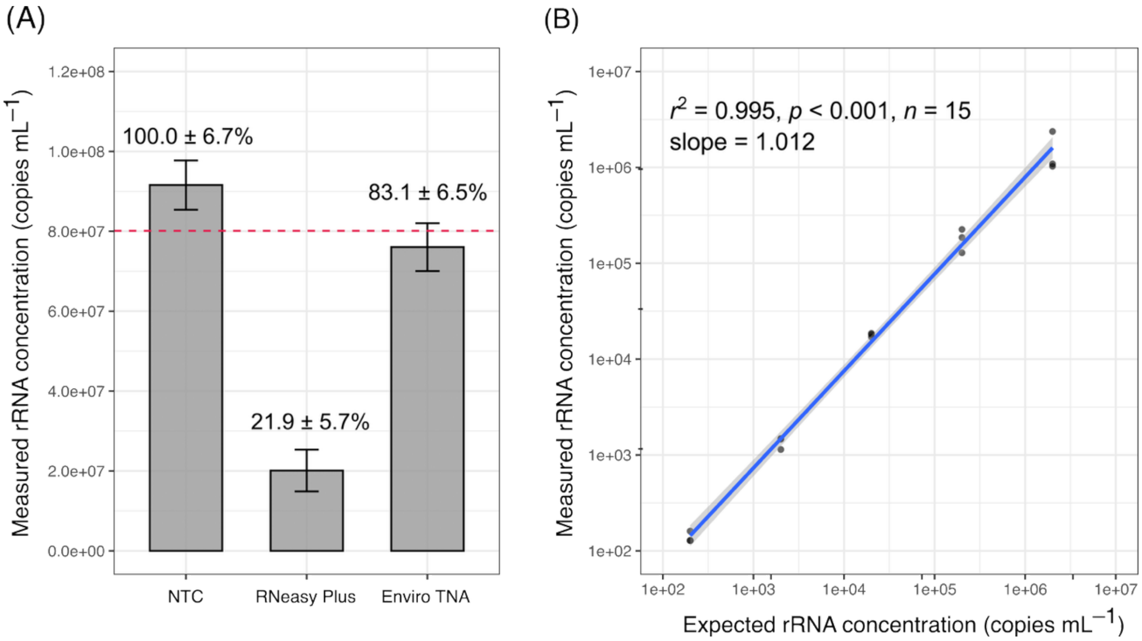

Figure S1. Performance of RNA extraction methods used. (A) Comparison of spiked-in rRNA concentrations (mean and SD, n = 3) recovered by two extraction kits and without extraction (i.e., non-treated control, NTC). The red dashed line denotes the expected values in the product data sheet. The value on top of each bar indicates the extraction efficiency calculated by comparing it with the NTC (mean and SD, n = 3). (B) Effect of spiked-in rRNA concentration on the extraction performance of the cell-free RNA extraction kit (Wizard Enviro TNA Kit). The linearity was evaluated in a 10-fold dilution series ranging from 2.0×10^2 to 2.0×10^6 copy mL^{-1} (log-log linear regression: $r^2 = 0.995$, $p < 0.001$, $n = 15$, the slope of 1.012 between the measured and expected values).

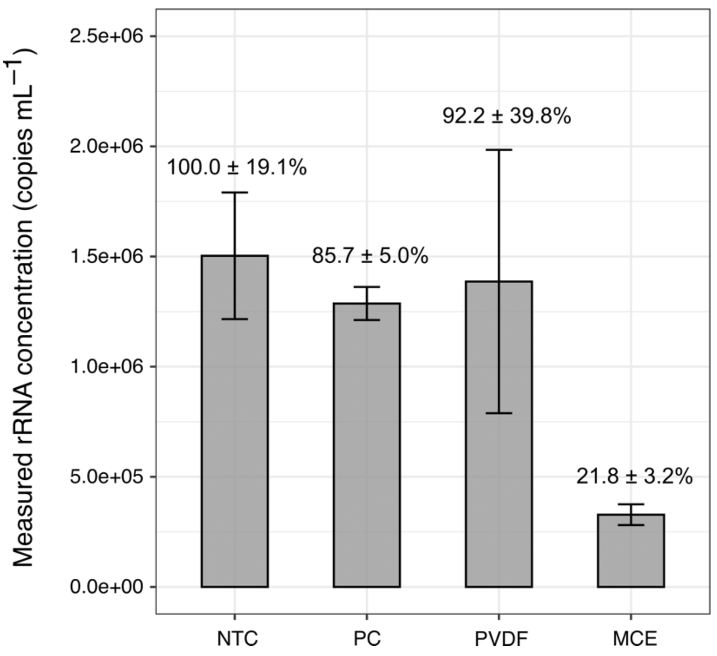

Figure S2. Performance of membrane filters used. Comparison of adsorption properties for ribosome (mean and SD, n = 3) with three filters made of polycarbonate (PC), polyvinylidene difluoride (PVDF), and mixed cellulose esters (MCE). The value on top of each bar indicates the recovery rate (mean and SD, n = 3) calculated by comparing it with the non-treated control (NTC).

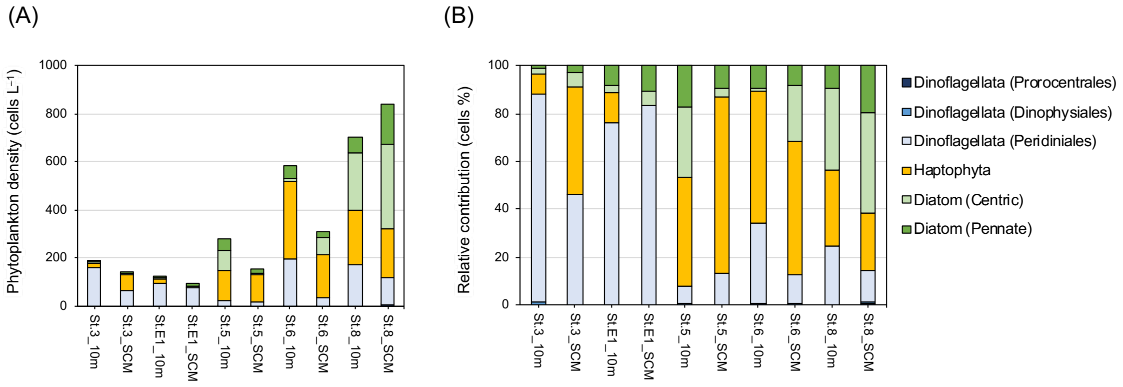

Figure S3. Phytoplankton cell counts were determined using light microscopy. (A) Abundance and (B) relative contributions of major phytoplankton in the epipelagic layer.
